# Dicaesium magnesium bis­(dihydrogen phosphate(V)) dihydrate

**DOI:** 10.1107/S1600536808042384

**Published:** 2008-12-17

**Authors:** Rachid Essehli, Brahim El Bali, Mohammed Lachkar, Michael Bolte

**Affiliations:** a Department of Chemistry, Faculty of Sciences, University Mohammed 1st, PO Box 717, 60 000 Oujda, Morocco; b LIMOM, Faculty of Sciences, University Sidi Mohamed Ben Abdellah, PO Box 1796 (Atlas), 30000 Fez, Morocco; c Institut für Anorganische Chemie, J. W. Goethe-Universität Frankfurt, Max-von-Laue-Strasse 7, 60438 Frankfurt/Main, Germany

## Abstract

The title compound, Cs_2_Mg(H_2_P_2_O_7_)_2_·2H_2_O, is isostructural with the related known isoformular phosphates. The crystal framework consists of corner-sharing MgO_6_ and H_2_P_2_O_7_ polyhedra, leading to tunnels parallel to the *b*-axis direction in which Cs^+^ ions are located. The H_2_P_2_O_7_ unit shows a bent eclipsed conformation. The Mg^2+^ ion lies on an inversion center. The water molecules form hydrogen bonds to O atoms of two different dihydrogenphosphate ions, which are further hydrogen bonded to symmetry-equivalent dihydrogenphosphate ions.

## Related literature

For isostructural phosphates, see: Capitelli *et al.* (2004 ▶), (NH_4_)_2_Mn(H_2_P_2_O_7_)_2_·2H_2_O; Essehli *et al.* (2005*a* ▶), (NH_4_)_2_Zn(H_2_P_2_O_7_)_2_·2H_2_O; Essehli *et al.* (2005*b* ▶), (NH_4_)_2_Ni(H_2_P_2_O_7_)_2_·2H_2_O; Essehli *et al.* (2005*c* ▶), (NH_4_)_2_Co(H_2_P_2_O_7_)_2_·2H_2_O; Tahiri *et al.* (2004 ▶), K_2_Ni(H_2_P_2_O_7_)_2_·2H_2_O; Tahiri *et al.* (2003 ▶), K_2_Zn(H_2_P_2_O_7_)_2_·2H_2_O; Harcharras *et al.* (2003 ▶), K_2_Mg(H_2_P_2_O_7_)_2_·2H_2_O. For the biological activity of inorganic acidic diphos­phates containing HP_2_O_7_^3−^ or H_2_P_2_O_7_^2−^ anions, see: Andreeva *et al.* (2001 ▶). 

## Experimental

### 

#### Crystal data

Cs_2_Mg(H_2_P_2_O_7_)_2_·2H_2_O
*M* *_r_* = 678.07
Triclinic, 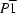

*a* = 7.0935 (15) Å
*b* = 7.4606 (15) Å
*c* = 8.0230 (15) Å
α = 83.776 (16)°
β = 68.558 (15)°
γ = 87.850 (17)°
*V* = 392.87 (14) Å^3^
*Z* = 1
Mo *K*α radiation
μ = 5.17 mm^−1^
*T* = 173 (2) K
0.19 × 0.15 × 0.08 mm

#### Data collection

Stoe IPDSII two-circle diffractometer
Absorption correction: multi-scan (*MULABS*; Spek, 2003 ▶; Blessing, 1995 ▶) *T*_min_ = 0.440, *T*_max_ = 0.683
3316 measured reflections
1420 independent reflections
1245 reflections with *I* > 2σ(*I*)
*R*_int_ = 0.082

#### Refinement

*R*[*F* ^2^ > 2σ(*F* ^2^)] = 0.048
*wR*(*F* ^2^) = 0.125
*S* = 1.02
1420 reflections
115 parameters
3 restraints
H atoms treated by a mixture of independent and constrained refinement
Δρ_max_ = 2.00 e Å^−3^
Δρ_min_ = −2.73 e Å^−3^

### 

Data collection: *X-AREA* (Stoe & Cie, 2001 ▶); cell refinement: *X-AREA*; data reduction: *X-AREA*; program(s) used to solve structure: *SHELXS97* (Sheldrick, 2008 ▶); program(s) used to refine structure: *SHELXL97* (Sheldrick, 2008 ▶); molecular graphics: *XP* in *SHELXTL-Plus* (Sheldrick, 2008 ▶); software used to prepare material for publication: *SHELXL97*.

## Supplementary Material

Crystal structure: contains datablocks global, I. DOI: 10.1107/S1600536808042384/br2088sup1.cif

Structure factors: contains datablocks I. DOI: 10.1107/S1600536808042384/br2088Isup2.hkl

Additional supplementary materials:  crystallographic information; 3D view; checkCIF report

## Figures and Tables

**Table 1:** Hydrogen-bond geometry (Å, °)

*D*—H⋯*A*	*D*—H	H⋯*A*	*D*⋯*A*	*D*—H⋯*A*
O1*W*—H1*WA*⋯O1^i^	0.839 (10)	2.01 (4)	2.804 (8)	158 (9)
O1*W*—H1*WB*⋯O6^ii^	0.839 (10)	1.97 (3)	2.778 (9)	162 (9)
O3—H3⋯O6^ii^	0.84	1.72	2.551 (8)	172
O7—H7⋯O1^iii^	0.84	1.71	2.518 (8)	159

Symmetry codes: (i) 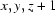; (ii) 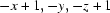; (iii) 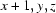.

**Fig. 1.**
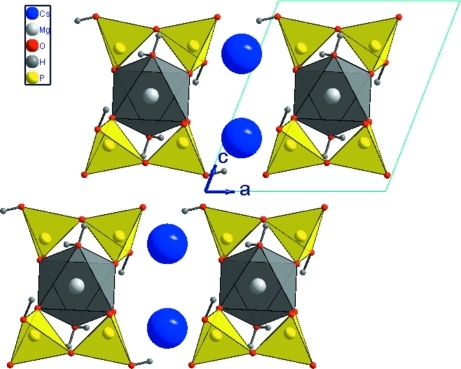
Crystal structure of Cs2Mn(H2P2O7)2.2H2O viewed along b direction.

**Fig. 2.**
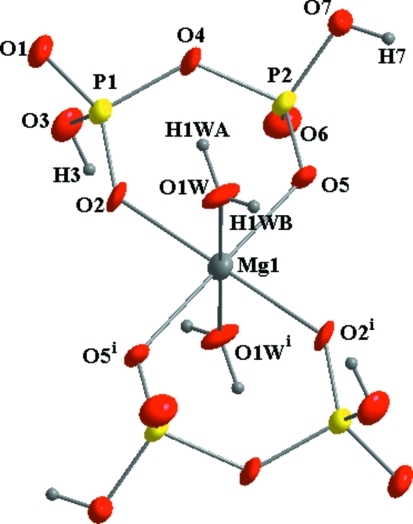
Mg coordination in Cs2Mn(H2P2O7)2.2H2O. Displacement ellipsoids are drawn at the 50% probability level. [Symmetry code: (i) - x,- y, - z.

## supplementary crystallographic information

### Comment

Inorganic acidic diphosphates containing HP_2_O_7_ or H_2_P_2_O_7_ hold important biochemical activities, such as inhibitors of human immunodeficiency enzymes as reported by Andreeva *et al.* (2001). In the framework of our systematic research on these phosphates, we report on the new compound Cs_2_Mg(H_2_P_2_O_7_)_2_.2H_2_O. Detailed studies on structure determinations of such phosphates are available in related crystallography literature.

The crystal packing of Cs_2_Mg(H_2_P_2_O_7_)_2_.2H_2_O is a 3D network made upon edges sharing [MgO_6_] octahedra and dihydrogendiphosphate [H_2_P_2_O_7_]. These delimite tunnels along *b* direction, where  Cs^+^ ions are located. A projection onto ac-plan is depicted on Fig. 1.

Mg^2+^ cation sites are on inversion center. It is coordinated by four O atoms from two bidendate [H_2_P_2_O_7_] groups and two remaining O atoms from water molecule (Fig. 2).

H_2_P_2_O_7_ shows bent eclipsed conformation. Distances and angles in [MgO_6_] and [H_2_P_2_O_7_] are as usual as in related phosphates structures. The [MgO_6_] are isolated in the structure, with an Mg-Mg distance over 7 Å.

### Experimental

Crystals of Cs_2_Mn(H_2_P_2_O_7_)_2_.2H_2_O were grown at room temperature by slow evaporation from water-ethanol (80/20) of aqueous solution containing a stoichiometric the mixture : MgCl_2_.6H_2_O (0.231mg, 1mmol), Cs_2_CO_3_ (0.24mg, 1mmol), and K_4_P_2_O_7_ (0.5mg, 1mmol). The solution was stirred for two hours at leaved to stand at room temperature. Crystals suitable for X-ray analysis were formed after few days.

### Refinement

All H atoms were located in a difference map. The water H atoms were refined with the O-H bonds restrained to 0.84 (1)Å and the H···H distances restrained to 1.4 (1)Å and with fixed individual displacement parameters [U(H) = 1.2 U_eq_(O)]. The H atoms of the hydroxyl groups bonded to P were refined using a riding model with O-H = 0.84Å, U(H) = 1.2 U_eq_(O) and P-O-H = 109.5 °.

### Crystal data

**Table:**

Cs_2_Mg(H_2_P_2_O_7_)_2_·2H_2_O	*Z* = 1
*M**_r_* = 678.07	*F*(000) = 318
Triclinic, *P*1	*D*_x_ = 2.866 Mg m^−^^3^
Hall symbol: -P 1	Mo *K*α radiation, λ = 0.71073 Å
*a* = 7.0935 (15) Å	Cell parameters from 3316 reflections
*b* = 7.4606 (15) Å	θ = 3.7–25.5°
*c* = 8.0230 (15) Å	µ = 5.17 mm^−^^1^
α = 83.776 (16)°	*T* = 173 K
β = 68.558 (15)°	Plate, colourless
γ = 87.850 (17)°	0.19 × 0.15 × 0.08 mm
*V* = 392.87 (14) Å^3^	

### Data collection

**Table:**

Stoe IPDSII two-circle diffractometer	1420 independent reflections
Radiation source: fine-focus sealed tube	1245 reflections with *I* > 2σ(*I*)
graphite	*R*_int_ = 0.082
ω scans	θ_max_ = 25.3°, θ_min_ = 3.7°
Absorption correction: multi-scan (*MULABS*; Spek, 2003; Blessing, 1995)	*h* = −8→8
*T*_min_ = 0.440, *T*_max_ = 0.683	*k* = −8→8
3316 measured reflections	*l* = −9→9

### Refinement

**Table:**

Refinement on *F*^2^	Secondary atom site location: difference Fourier map
Least-squares matrix: full	Hydrogen site location: inferred from neighbouring sites
*R*[*F*^2^ > 2σ(*F*^2^)] = 0.048	H atoms treated by a mixture of independent and constrained refinement
*wR*(*F*^2^) = 0.125	*w* = 1/[σ^2^(*F*_o_^2^) + (0.084*P*)^2^] where *P* = (*F*_o_^2^ + 2*F*_c_^2^)/3
*S* = 1.02	(Δ/σ)_max_ < 0.001
1420 reflections	Δρ_max_ = 2.00 e Å^−^^3^
115 parameters	Δρ_min_ = −2.73 e Å^−^^3^
3 restraints	Extinction correction: *SHELXL97* (Sheldrick, 2008), Fc^*^=kFc[1+0.001xFc^2^λ^3^/sin(2θ)]^-1/4^
Primary atom site location: structure-invariant direct methods	Extinction coefficient: 0.011 (3)

### Special details

**Table:**

Geometry. All esds (except the esd in the dihedral angle between two l.s. planes) are estimated using the full covariance matrix. The cell esds are taken into account individually in the estimation of esds in distances, angles and torsion angles; correlations between esds in cell parameters are only used when they are defined by crystal symmetry. An approximate (isotropic) treatment of cell esds is used for estimating esds involving l.s. planes.
Refinement. Refinement of F^2^ against ALL reflections. The weighted R-factor wR and goodness of fit S are based on F^2^, conventional R-factors R are based on F, with F set to zero for negative F^2^. The threshold expression of F^2^ > 2sigma(F^2^) is used only for calculating R-factors(gt) etc. and is not relevant to the choice of reflections for refinement. R-factors based on F^2^ are statistically about twice as large as those based on F, and R- factors based on ALL data will be even larger.

### Fractional atomic coordinates and isotropic or equivalent isotropic displacement parameters (Å^2^)

**Table:**

	*x*	*y*	*z*	*U*_iso_*/*U*_eq_	
Cs1	0.09044 (7)	0.70386 (7)	0.27841 (6)	0.0186 (3)	
Mg1	0.5000	0.5000	0.5000	0.0137 (8)	
O1W	0.4211 (9)	0.2941 (8)	0.7146 (7)	0.0195 (14)	
H1WA	0.413 (14)	0.319 (13)	0.817 (6)	0.023*	
H1WB	0.355 (13)	0.201 (8)	0.719 (12)	0.023*	
P1	0.3736 (3)	0.2528 (3)	0.2388 (3)	0.0129 (5)	
P2	0.7759 (3)	0.1985 (3)	0.2518 (3)	0.0130 (5)	
O1	0.3041 (8)	0.3164 (9)	0.0855 (8)	0.0190 (13)	
O2	0.3344 (8)	0.3767 (8)	0.3829 (7)	0.0151 (12)	
O3	0.2791 (9)	0.0628 (8)	0.3238 (8)	0.0181 (13)	
H3	0.2828	0.0417	0.4276	0.022*	
O4	0.6144 (8)	0.2214 (8)	0.1485 (7)	0.0162 (12)	
O5	0.7572 (8)	0.3505 (8)	0.3636 (7)	0.0155 (12)	
O6	0.7434 (9)	0.0105 (8)	0.3514 (8)	0.0216 (14)	
O7	0.9737 (9)	0.2074 (9)	0.0826 (8)	0.0201 (14)	
H7	1.0655	0.2565	0.1046	0.024*	

### Atomic displacement parameters (Å^2^)

**Table:**

	*U*^11^	*U*^22^	*U*^33^	*U*^12^	*U*^13^	*U*^23^
Cs1	0.0193 (4)	0.0231 (4)	0.0164 (4)	0.0023 (2)	−0.0101 (2)	−0.0027 (2)
Mg1	0.0124 (18)	0.018 (2)	0.0124 (18)	0.0022 (15)	−0.0069 (15)	−0.0011 (15)
O1W	0.030 (4)	0.022 (3)	0.010 (3)	−0.003 (3)	−0.012 (3)	−0.002 (2)
P1	0.0121 (10)	0.0171 (11)	0.0121 (10)	0.0017 (8)	−0.0073 (8)	−0.0031 (8)
P2	0.0131 (10)	0.0190 (11)	0.0104 (9)	0.0015 (8)	−0.0080 (8)	−0.0035 (8)
O1	0.016 (3)	0.029 (3)	0.017 (3)	−0.001 (2)	−0.012 (2)	−0.002 (3)
O2	0.013 (3)	0.021 (3)	0.017 (3)	0.000 (2)	−0.012 (2)	−0.006 (2)
O3	0.022 (3)	0.017 (3)	0.021 (3)	−0.005 (2)	−0.012 (3)	−0.005 (2)
O4	0.013 (3)	0.027 (3)	0.014 (3)	0.003 (2)	−0.011 (2)	−0.005 (2)
O5	0.014 (3)	0.025 (3)	0.012 (3)	0.002 (2)	−0.008 (2)	−0.009 (2)
O6	0.027 (3)	0.022 (3)	0.017 (3)	−0.002 (3)	−0.010 (3)	−0.001 (2)
O7	0.012 (3)	0.037 (4)	0.014 (3)	−0.001 (3)	−0.006 (2)	−0.008 (3)

### Geometric parameters (Å, °)

**Table:**

Cs1—O7^i^	3.092 (6)	O1W—Cs1^iv^	3.608 (6)
Cs1—O3^ii^	3.151 (6)	O1W—H1WA	0.839 (10)
Cs1—O2	3.155 (6)	O1W—H1WB	0.839 (10)
Cs1—O6^iii^	3.233 (7)	P1—O2	1.498 (6)
Cs1—O2^iv^	3.259 (6)	P1—O1	1.510 (6)
Cs1—O4^i^	3.295 (5)	P1—O3	1.566 (6)
Cs1—O5^v^	3.401 (5)	P1—O4	1.612 (6)
Cs1—O5^vi^	3.450 (6)	P1—Cs1^iv^	4.100 (2)
Cs1—O1	3.461 (6)	P2—O5	1.493 (6)
Cs1—O1W^v^	3.486 (6)	P2—O6	1.518 (6)
Cs1—O1W^iv^	3.608 (6)	P2—O7	1.553 (6)
Cs1—P1	3.836 (2)	P2—O4	1.637 (5)
Mg1—O2	2.046 (5)	P2—Cs1^i^	3.995 (2)
Mg1—O2^v^	2.046 (5)	O2—Cs1^iv^	3.259 (6)
Mg1—O5^v^	2.103 (6)	O3—Cs1^viii^	3.151 (6)
Mg1—O5	2.103 (6)	O3—H3	0.8400
Mg1—O1W^v^	2.103 (5)	O4—Cs1^i^	3.295 (5)
Mg1—O1W	2.103 (5)	O5—Cs1^v^	3.401 (5)
Mg1—Cs1^v^	4.0953 (9)	O5—Cs1^vii^	3.450 (6)
Mg1—Cs1^vii^	4.1789 (11)	O6—Cs1^ix^	3.233 (6)
Mg1—Cs1^iv^	4.1790 (11)	O7—Cs1^i^	3.092 (6)
O1W—Cs1^v^	3.486 (6)	O7—H7	0.8400
			
O7^i^—Cs1—O3^ii^	103.03 (16)	O5—Mg1—Cs1^v^	56.01 (15)
O7^i^—Cs1—O2	127.15 (16)	O1W^v^—Mg1—Cs1^v^	121.65 (16)
O3^ii^—Cs1—O2	108.02 (15)	O1W—Mg1—Cs1^v^	58.35 (16)
O7^i^—Cs1—O6^iii^	74.51 (15)	O2—Mg1—Cs1	48.97 (16)
O3^ii^—Cs1—O6^iii^	72.09 (16)	O2^v^—Mg1—Cs1	131.03 (16)
O2—Cs1—O6^iii^	155.89 (14)	O5^v^—Mg1—Cs1	56.01 (15)
O7^i^—Cs1—O2^iv^	112.39 (14)	O5—Mg1—Cs1	123.99 (15)
O3^ii^—Cs1—O2^iv^	108.69 (15)	O1W^v^—Mg1—Cs1	58.35 (16)
O2—Cs1—O2^iv^	96.77 (14)	O1W—Mg1—Cs1	121.65 (16)
O6^iii^—Cs1—O2^iv^	61.80 (15)	Cs1^v^—Mg1—Cs1	180.000 (7)
O7^i^—Cs1—O4^i^	44.14 (14)	O2—Mg1—Cs1^vii^	130.16 (15)
O3^ii^—Cs1—O4^i^	84.93 (15)	O2^v^—Mg1—Cs1^vii^	49.84 (16)
O2—Cs1—O4^i^	97.36 (14)	O5^v^—Mg1—Cs1^vii^	124.62 (16)
O6^iii^—Cs1—O4^i^	106.59 (14)	O5—Mg1—Cs1^vii^	55.38 (16)
O2^iv^—Cs1—O4^i^	156.16 (13)	O1W^v^—Mg1—Cs1^vii^	59.70 (16)
O7^i^—Cs1—O5^v^	169.36 (14)	O1W—Mg1—Cs1^vii^	120.30 (16)
O3^ii^—Cs1—O5^v^	68.88 (15)	Cs1^v^—Mg1—Cs1^vii^	61.976 (19)
O2—Cs1—O5^v^	52.76 (15)	Cs1—Mg1—Cs1^vii^	118.02 (2)
O6^iii^—Cs1—O5^v^	108.24 (14)	O2—Mg1—Cs1^iv^	49.84 (16)
O2^iv^—Cs1—O5^v^	77.41 (13)	O2^v^—Mg1—Cs1^iv^	130.16 (16)
O4^i^—Cs1—O5^v^	126.34 (13)	O5^v^—Mg1—Cs1^iv^	55.38 (16)
O7^i^—Cs1—O5^vi^	86.75 (14)	O5—Mg1—Cs1^iv^	124.62 (16)
O3^ii^—Cs1—O5^vi^	160.22 (15)	O1W^v^—Mg1—Cs1^iv^	120.30 (16)
O2—Cs1—O5^vi^	78.07 (14)	O1W—Mg1—Cs1^iv^	59.70 (16)
O6^iii^—Cs1—O5^vi^	94.58 (15)	Cs1^v^—Mg1—Cs1^iv^	118.024 (19)
O2^iv^—Cs1—O5^vi^	51.54 (14)	Cs1—Mg1—Cs1^iv^	61.98 (2)
O4^i^—Cs1—O5^vi^	113.40 (13)	Cs1^vii^—Mg1—Cs1^iv^	180.0
O5^v^—Cs1—O5^vi^	103.09 (12)	Mg1—O1W—Cs1^v^	90.75 (19)
O7^i^—Cs1—O1	82.39 (15)	Mg1—O1W—Cs1^iv^	90.08 (18)
O3^ii^—Cs1—O1	132.65 (15)	Cs1^v^—O1W—Cs1^iv^	178.20 (18)
O2—Cs1—O1	45.25 (13)	Mg1—O1W—H1WA	119 (7)
O6^iii^—Cs1—O1	150.16 (14)	Cs1^v^—O1W—H1WA	72 (7)
O2^iv^—Cs1—O1	112.32 (15)	Cs1^iv^—O1W—H1WA	106 (7)
O4^i^—Cs1—O1	66.40 (15)	Mg1—O1W—H1WB	124 (6)
O5^v^—Cs1—O1	97.95 (14)	Cs1^v^—O1W—H1WB	125 (7)
O5^vi^—Cs1—O1	65.07 (13)	Cs1^iv^—O1W—H1WB	55 (7)
O7^i^—Cs1—O1W^v^	119.58 (14)	H1WA—O1W—H1WB	113 (8)
O3^ii^—Cs1—O1W^v^	59.65 (15)	O2—P1—O1	116.7 (4)
O2—Cs1—O1W^v^	51.99 (14)	O2—P1—O3	110.1 (3)
O6^iii^—Cs1—O1W^v^	131.44 (15)	O1—P1—O3	108.9 (3)
O2^iv^—Cs1—O1W^v^	128.02 (13)	O2—P1—O4	108.7 (3)
O4^i^—Cs1—O1W^v^	75.64 (13)	O1—P1—O4	106.0 (3)
O5^v^—Cs1—O1W^v^	50.71 (13)	O3—P1—O4	106.0 (3)
O5^vi^—Cs1—O1W^v^	130.02 (14)	O2—P1—Cs1	52.5 (2)
O1—Cs1—O1W^v^	76.60 (14)	O1—P1—Cs1	64.3 (3)
O7^i^—Cs1—O1W^iv^	61.98 (13)	O3—P1—Cs1	126.4 (2)
O3^ii^—Cs1—O1W^iv^	119.49 (15)	O4—P1—Cs1	127.4 (2)
O2—Cs1—O1W^iv^	128.11 (13)	O2—P1—Cs1^iv^	46.6 (2)
O6^iii^—Cs1—O1W^iv^	47.53 (15)	O1—P1—Cs1^iv^	110.0 (2)
O2^iv^—Cs1—O1W^iv^	50.41 (12)	O3—P1—Cs1^iv^	69.8 (2)
O4^i^—Cs1—O1W^iv^	105.98 (12)	O4—P1—Cs1^iv^	143.1 (2)
O5^v^—Cs1—O1W^iv^	127.65 (13)	Cs1—P1—Cs1^iv^	64.84 (4)
O5^vi^—Cs1—O1W^iv^	50.22 (14)	O5—P2—O6	116.1 (3)
O1—Cs1—O1W^iv^	104.72 (14)	O5—P2—O7	112.9 (3)
O1W^v^—Cs1—O1W^iv^	178.20 (18)	O6—P2—O7	110.9 (4)
O7^i^—Cs1—P1	105.41 (12)	O5—P2—O4	110.8 (3)
O3^ii^—Cs1—P1	122.63 (12)	O6—P2—O4	106.4 (3)
O2—Cs1—P1	22.12 (11)	O7—P2—O4	98.0 (3)
O6^iii^—Cs1—P1	164.02 (12)	O5—P2—Cs1^i^	120.3 (2)
O2^iv^—Cs1—P1	104.76 (11)	O6—P2—Cs1^i^	123.5 (3)
O4^i^—Cs1—P1	82.42 (11)	O4—P2—Cs1^i^	53.5 (2)
O5^v^—Cs1—P1	74.87 (11)	P1—O1—Cs1	92.5 (3)
O5^vi^—Cs1—P1	69.55 (10)	P1—O2—Mg1	137.3 (4)
O1—Cs1—P1	23.15 (9)	P1—O2—Cs1	105.4 (3)
O1W^v^—Cs1—P1	63.00 (11)	Mg1—O2—Cs1	101.8 (2)
O1W^iv^—Cs1—P1	117.80 (10)	P1—O2—Cs1^iv^	113.8 (3)
O2—Mg1—O2^v^	179.999 (1)	Mg1—O2—Cs1^iv^	101.5 (2)
O2—Mg1—O5^v^	89.5 (2)	Cs1—O2—Cs1^iv^	83.23 (14)
O2^v^—Mg1—O5^v^	90.5 (2)	P1—O3—Cs1^viii^	148.3 (3)
O2—Mg1—O5	90.5 (2)	P1—O3—H3	109.5
O2^v^—Mg1—O5	89.5 (2)	Cs1^viii^—O3—H3	102.2
O5^v^—Mg1—O5	179.999 (1)	P1—O4—P2	126.8 (4)
O2—Mg1—O1W^v^	89.7 (2)	P1—O4—Cs1^i^	128.3 (3)
O2^v^—Mg1—O1W^v^	90.3 (2)	P2—O4—Cs1^i^	103.0 (2)
O5^v^—Mg1—O1W^v^	89.1 (2)	P2—O5—Mg1	129.5 (3)
O5—Mg1—O1W^v^	90.9 (2)	P2—O5—Cs1^v^	120.1 (3)
O2—Mg1—O1W	90.3 (2)	Mg1—O5—Cs1^v^	93.14 (17)
O2^v^—Mg1—O1W	89.7 (2)	P2—O5—Cs1^vii^	127.4 (3)
O5^v^—Mg1—O1W	90.9 (2)	Mg1—O5—Cs1^vii^	94.5 (2)
O5—Mg1—O1W	89.1 (2)	Cs1^v^—O5—Cs1^vii^	76.91 (12)
O1W^v^—Mg1—O1W	180.0 (3)	P2—O6—Cs1^ix^	123.7 (3)
O2—Mg1—Cs1^v^	131.03 (16)	P2—O7—Cs1^i^	114.5 (3)
O2^v^—Mg1—Cs1^v^	48.97 (16)	P2—O7—H7	109.5
O5^v^—Mg1—Cs1^v^	123.99 (15)	Cs1^i^—O7—H7	122.0

Symmetry codes: (i) −*x*+1, −*y*+1, −*z*; (ii) *x*, *y*+1, *z*; (iii) *x*−1, *y*+1, *z*; (iv) −*x*, −*y*+1, −*z*+1; (v) −*x*+1, −*y*+1, −*z*+1; (vi) *x*−1, *y*, *z*; (vii) *x*+1, *y*, *z*; (viii) *x*, *y*−1, *z*; (ix) *x*+1, *y*−1, *z*.

### Hydrogen-bond geometry (Å, °)

**Table:**

*D*—H···*A*	*D*—H	H···*A*	*D*···*A*	*D*—H···*A*
O1W—H1WA···O1^x^	0.84 (1)	2.01 (4)	2.804 (8)	158 (9)
O1W—H1WB···O6^xi^	0.84 (1)	1.97 (3)	2.778 (9)	162 (9)
O3—H3···O6^xi^	0.84	1.72	2.551 (8)	172
O7—H7···O1^vii^	0.84	1.71	2.518 (8)	159

Symmetry codes: (x) *x*, *y*, *z*+1; (xi) −*x*+1, −*y*, −*z*+1; (vii) *x*+1, *y*, *z*.
